# Medical Transport of Children with Complex Chronic Conditions

**DOI:** 10.1155/2012/837020

**Published:** 2012-01-18

**Authors:** Carlos F. Lerner, Robert B. Kelly, Leslie J. Hamilton, Thomas S. Klitzner

**Affiliations:** Department of Pediatrics, Mattel Children's Hospital UCLA, David Geffen School of Medicine at UCLA, Los Angeles, CA 90095, USA

## Abstract

One of the most notable trends in child health has been the increase in the number of children with special health care needs, including those with complex chronic conditions. Care of these children accounts for a growing fraction of health care resources. We examine recent developments in health care, especially with regard to medical transport and prehospital care, that have emerged to adapt to this remarkable demographic trend. One such development is the focus on care coordination, including the dissemination of the patient-centered medical home concept. In the prehospital setting, the need for greater coordination has catalyzed the development of the emergency information form. Training programs for prehospital providers now incorporate specific modules for children with complex conditions. Another notable trend is the shift to a family-centered model of care. We explore efforts toward regionalization of care, including the development of specialized pediatric transport teams, and conclude with recommendations for a research agenda.

## 1. Introduction

Since the National Academy of Sciences issued its seminal white paper in 1966, “Accidental Death and Disability” [1], which provided the impetus for the development of the modern emergency medical services in the United States, there have been profound changes in the health care needs of the American population. In child health, while injuries remain a significant contributor to pediatric morbidity and mortality, one of the most notable trends has been an increase in the number of children with chronic conditions [2–5]. In this review, we examine recent developments in health care, with a particular focus on medical transport and prehospital care, that have emerged in recent decades to adapt to this remarkable increase in children with complex medical conditions.

## 2. Methodology

We summarize trends in the care of children with complex chronic conditions in the United States, and we review the published literature on the transport and prehospital care of these children. In areas where there is little published evidence, we describe the relevant experience of our institution. Finally, we identify gaps in the published literature to generate a suggested research agenda.

## 3. Results and Discussion

### 3.1. Growing Impact of Children with Complex Chronic Conditions

Advances in neonatology, critical care, emergency medicine, and many other areas of pediatric medicine have resulted in increased survival of children with complex chronic conditions. Extremely premature infants, children with complex congenital heart disease, and children with rare genetic or metabolic conditions are surviving in greater numbers and living longer, frequently into adulthood [6–11]. These children typically have multisystem diseases, complex medication regimens, and sometimes utilize an array of medical technologies such as home ventilators or gastrostomy tubes [7, 9]. Care for this population of children accounts for a significant and growing fraction of health care resources, and places increased demands at every level of the health care system. In 2006, children with complex chronic conditions accounted for 26.1% of pediatric hospital days and 43.2% of charges in the United States [12]. In an analysis from a single health plan, children with chronic conditions, representing 10% of the population, accounted for nearly 50% of total medical charges; those with catastrophic or multiple significant chronic medical conditions (excluding cancer) represented 0.5% of the total population and over 15% of total medical charges [13]. A study of unscheduled admissions to a regional pediatric intensive care unit noted that technology-assisted children comprised <0.5% of the population and 14% of admissions [14].

 In the prehospital context, advances in technology and changing cultural norms have allowed more children with complex or technology-dependent chronic disease to live at home or in community settings, further increasing their demand for prehospital services. Published data describing these trends, however, remain quite limited and outdated. Suruda et al. review emergency medical service (EMS) run records between 1991 and 1992 in Utah [15]. Using various definitions of children with special health care needs (CSHCNs), these authors noted that between 23% and 78% of EMS runs for CSHCN were for interfacility transports. They also noted that these children were more likely to receive advanced life support and prehospital procedures. Spaite et al. analyze EMS responses for CSHCN in Tucson, Arizona in 1997-98 [16]. They found that children accounted for 18% of all EMS responses, but only 2% of responses were for CSHCN. These studies are limited by their small size, by methodological challenges of defining CSHCN, and by their focus on single geographic regions. Nevertheless, well-documented population-level trends [6–11], and data from other parts of the health care system [12–14], justify a continued focus on expanding capacity to provide quality prehospital care to children with complex conditions.

### 3.2. Increased Focus on Care Coordination and Integration

Typically, children with complex chronic conditions receive services from multiple physicians and other health care providers, including advanced practice nurses, physical therapists, occupational therapists, physiatrists, and pharmacists as well as various community and school-based agencies. They depend on complex medication regimens, care plans, and various medical technologies. Enhancing care coordination has become a central focus of efforts to improve the care of these children. The Medical Home, a model of primary care delivery first introduced within pediatrics, has recently been endorsed by the major American primary care organizations as a model for quality primary care [17]. Robust care coordination is one of the pillars of the Medical Home [17, 18].

 In the prehospital setting, the perceived need for improved care coordination has catalyzed the development of the emergency information form (EIF). A readily available, concise, accurate, and updated summary of the child's medical record can facilitate the provision of quality care by prehospital providers. In 1999, a joint policy statement by the American Academy of Pediatrics and the American College of Emergency Physicians introduced the emergency information form (EIF), a single-sheet medical summary of essential medical information for the initial treatment of CSHCN [19]. In 2010, these organizations updated their EIF recommendations, noting that the EIF has been underused due to lack of awareness among health care providers and families, and the perception among many providers that completing such a document is time consuming and of limited usefulness [20]. The updated statement affirms that the completion of the EIF “should be the responsibility of the medical home primary care physician and specialty care providers for every child with special health care needs.” Additionally, the statement calls for the establishment of a central standardized electronic repository of EIFs. At present, however, we are unaware of any published data regarding adherence to these recommendations, aside from single-program descriptions, such as the Minnesota Emergency Medical Services for Children Information System, which provides a web-based repository of EIFs for infants and children with heart disease [21].

### 3.3. Enhanced Training for Prehospital Care and Transport of Children with Special Health Care Needs

Recognizing the increasing numbers of children with complex chronic conditions, and the increasing complexity of their care, training programs pertinent to prehospital care providers have incorporated instruction on the care of these children. Three well-established programs include specific modules for this population: the Pediatric Advanced Life Support (PALS) course (developed by the American Heart Association) [22], the Pediatric Education for Prehospital Professionals (PEPP) course (from the Academy of Pediatrics) [23], and the Advanced Pediatric Life Support (APLS) course (jointly presented by the American Academy of Pediatrics and the American College of Emergency Physicians) [24].

 Additionally, specific training programs have been developed for this population. The Center for Prehospital Pediatrics at Children's National Medical Center developed a continuing medical education curriculum, special children's outreach and prehospital education (SCOPE), which provides basic information on chronic medical conditions and an overview of commonly used medical technologies [25]. The investigators also published a resource template for the development of local emergency medical service protocols implementing the SCOPE program [26]. Similarly, two reports in the medical literature address the emergency medical management of technology-assisted children [27, 28]. Technologies reviewed in these reports include tracheostomies, apnea monitors, home ventilators, central venous catheters, enteral feeding tubes, colostomies/ileostomies, artificial pacemakers, and cerebrospinal fluid shunts.

 Another component of prehospital care of medically complex children involves the readiness of pediatric offices to manage medical emergencies and stabilize patients pending transport to higher level of care. The American Academy of Pediatrics has recently outlined recommendations for pediatric office emergencies [29]. Of utmost importance is the identification of a medical team leader. Ideally, this physician should have knowledge of basic pediatric critical care stabilization, particularly of airway and cardiac support. Pediatric Advanced Life Support certification is recommended. Recommended office equipment at a minimum includes an oxygen source, a nonrebreather mask, a bag-valve-mask resuscitator, suction, nebulizer, oropharyngeal airways, pulse oximeter, drug dose reference, rigid board, sphygmomanometer, splints, sterile dressings, epinephrine, and albuterol for inhalation [29]. For practices caring for children with complex conditions, we additionally suggest intravenous catheters, intraosseous needles, a cardiac monitor, an automated external defibrillator, atropine, adenosine, and amiodarone.

 Although evidence now strongly supports the use of simulation training in pediatric residency education [30], prehospital resuscitation training need not involve expensive computerized pediatric simulators. Simply organizing an office emergency plan, stocking essential airway equipment, purchasing basic cardiac medications, and regularly running mock scenarios can prepare office staff for decompensating medically complex patients. Data reported by Toback et al. demonstrate the increased confidence gained by ambulatory clinic personnel after a mock code initiative [31]. Mock scenarios should ideally establish roles for each member of the staff, including ancillary office personnel. Common roles during an emergency should include an individual who calls local emergency medical personnel, one who leads the resuscitation, one who assumes care of the airway, one who establishes vascular access, one who records medical interventions, and one who provides family support. Current evidence supports the inclusion of caregivers during inpatient cardiopulmonary resuscitation [32], and, by extension, we recommend this practice in the prehospital care setting.

### 3.4. Focus on Family-Centered Care

The shift to a family-centered model of care, in which families and medical providers comprise equal partners in the medical care of the child, has transformed the care of children with special health care needs [33]. Strong partnerships with families constitute another pillar of the medical home [17, 18]. Family-centered care for CSHCN has been associated with improvements in efficient use of services, health status, satisfaction, and access to care [34].

 In the prehospital setting, participation of the family (and, if available, the home health nurse) in the evaluation and management of the medically complex child has been recommended as the key to quality care [25, 27, 28]. In our experience at the Pediatric Medical Home Program at UCLA [35], the principal caregiver for the child is typically best suited to describe the child's baseline vital signs, to evaluate mental status and abilities, to assess the changes from baseline and the severity of the child's condition, to assist with manipulating and troubleshooting medical technology devices, and to suggest a course of treatment. We strongly endorse this family-centered approach based on clinical experience and extrapolation from findings in other areas of health care [34].

### 3.5. Regionalization of Care

Care for this highly complex population can require specialized skills and knowledge as well as intensive care coordination. The Patient-Centered Medical Home concept emphasizes enhancing the infrastructure of community pediatric practices to be able to better care for such children [17, 18]. An alternative approach to deliver such care has been the development of regional programs, frequently associated with academic medical centers, which centralize the primary care of highly medically complex children [35–37]. Such programs have been associated with decreased Emergency Department visits [35] and decreased inpatient lengths of stay [36].

**Figure 1:**
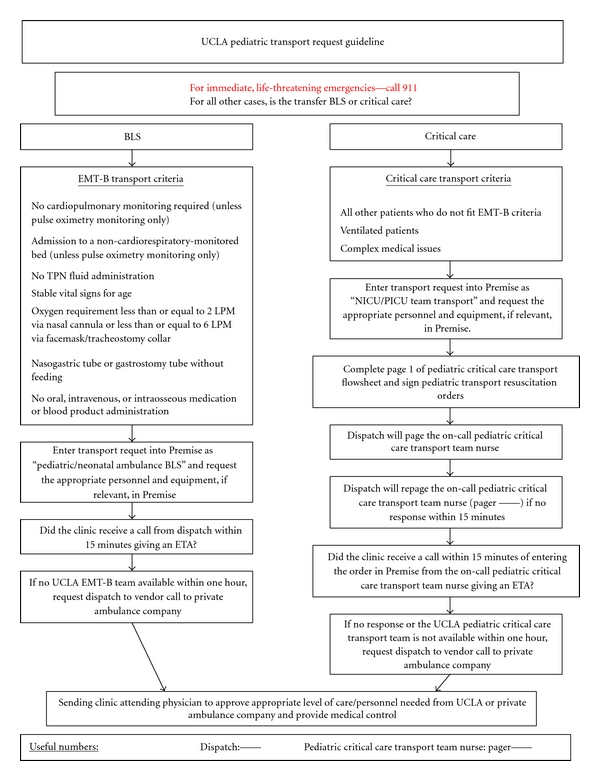
UCLA algorithm for transport of pediatric patients from medical offices to a higher level of care.

A parallel effort in the medical transport setting has been the development of specialized pediatric transport teams, particularly for the interfacility transport of children to higher levels of care. Specialized pediatric transport teams were found in one report to have fewer deaths and fewer unplanned events, including airway-related events, cardiopulmonary arrest, sustained hypotension, and loss of intravenous access [38]. When multiple options for the transport of pediatric patients are available, we recommend creating and disseminating an algorithm for the pediatric transport of medically complex patients. In our health system, for example, transport services can be provided by local emergency medical services, hospital-based emergency medical technicians (EMTs), or a specialized hospital-based pediatric transport team consisting of EMTs, a nurse, a respiratory therapist, and, if required, a pediatric physician. For immediate, life-threatening emergencies at one of our medical offices, local emergency medical services are called. For nonemergent responses, hospital-based EMTs independently transport any pediatric patient unless the patient meets certain exclusion criteria (Figure 1). In those situations, including patients with particularly complex care, our specialized pediatric transport team is dispatched. The sending physician serves as the medical control officer for the entire transport and must approve the appropriate level of care. All transport requests completed by our health system transport personnel are requested electronically, providing a timeline for legal and quality review, as needed.

Open and direct communication between sending and receiving medical teams ensures a smooth transfer of care. Centralized communication centers should be utilized to facilitate conference calls between all parties involved in the care of these complex patients. We recommend consulting the Guidelines for Air and Ground Transport of Neonatal and Pediatric Patients published by the American Academy of Pediatrics for further guidance on establishing such a transport system [39].

## 4. Conclusions: Setting a Research Agenda

A growing body of medical literature describes the increase in the number of children with complex chronic diseases, the impact of this increase on the health care system, and novel approaches to care for these children. As described in this review, however, minimal work in this area has been published that is specifically relevant to prehospital medicine and medical transport for this population. Research in a range of areas is urgently needed for prehospital medicine to adapt to this major demographic transformation. We highlight several suggested areas for further research.

**(1) Description of the Epidemiology of Prehospital Care for Children with Complex Chronic Conditions**

What is the resource utilization for prehospital and EMS services for children with complex chronic conditions, at the local, regional, and national level? How has this pattern changed over time? What are the characteristics of these children? What are the characteristics of prehospital responses for these patients?

**(2) Emergency Information Forms**

What are the optimal components of such forms? How widely are such forms being used? What are the barriers to their implementation? Can quality improvement efforts succeed in increasing their use? How can primary care medical homes support the delivery of quality prehospital care through the use of such forms or other mechanisms?

**(3) Training Programs for Prehospital Care and Transport of Children with Special Health Care Needs**

How widely accessible and utilized are such programs? What training program components lead to measurable improvement in the delivery of prehospital care to these children? What is the most effective way to manage specific technologies in the prehospital setting?

**(4) Specialized Pediatric Transport**

What is the optimal role of specialized pediatric transport teams? What is the cost and efficacy of such teams?

Medical care provided in the prehospital setting is a key component in the continuum of care for all patients, but particularly for children with chronic complex medical conditions. Understanding the answers to these questions is becoming increasingly important as the number of these children grows, and as their options continue to expand for living in their homes and communities.
